# Expression and Properties of the Highly Alkalophilic Phenylalanine Ammonia-Lyase of Thermophilic *Rubrobacter xylanophilus*

**DOI:** 10.1371/journal.pone.0085943

**Published:** 2014-01-27

**Authors:** Klaudia Kovács, Gergely Bánóczi, Andrea Varga, Izabella Szabó, András Holczinger, Gábor Hornyánszky, Imre Zagyva, Csaba Paizs, Beáta G. Vértessy, László Poppe

**Affiliations:** 1 Department of Organic Chemistry and Technology, Budapest University of Technology and Economics, Budapest, Hungary; 2 Institute of Enzymology, Research Centre for Natural Sciences of Hungarian Academy of Sciences, Budapest, Hungary; 3 Biocatalysis Research Group, Babeş-Bolyai University of Cluj-Napoca, Cluj-Napoca, Romania; 4 Department of Applied Biotechnology and Food Science, Budapest University of Technology and Economics, Budapest, Hungary; CNR, Italy

## Abstract

The sequence of a phenylalanine ammonia-lyase (PAL; EC: 4.3.1.24) of the thermophilic and radiotolerant bacterium *Rubrobacter xylanophilus* (*Rx*PAL) was identified by screening the genomes of bacteria for members of the phenylalanine ammonia-lyase family. A synthetic gene encoding the *Rx*PAL protein was cloned and overexpressed in *Escherichia coli* TOP 10 in a soluble form with an *N*-terminal His_6_-tag and the recombinant *Rx*PAL protein was purified by Ni-NTA affinity chromatography. The activity assay of *Rx*PAL with l-phenylalanine at various pH values exhibited a local maximum at pH 8.5 and a global maximum at pH 11.5. Circular dichroism (CD) studies showed that *Rx*PAL is associated with an extensive α-helical character (far UV CD) and two distinctive near-UV CD peaks. These structural characteristics were well preserved up to pH 11.0. The extremely high pH optimum of *Rx*PAL can be rationalized by a three-dimensional homology model indicating possible disulfide bridges, extensive salt-bridge formation and an excess of negative electrostatic potential on the surface. Due to these properties, *Rx*PAL may be a candidate as biocatalyst in synthetic biotransformations leading to unnatural l- or d-amino acids or as therapeutic enzyme in treatment of phenylketonuria or leukemia.

## Introduction

Enzymes are increasingly popular as efficient, clean and environmentally friendly catalysts in industrial applications ranging from additives to laundry detergents, as well as paper processing or in the synthesis of fine chemicals and diagnostic/research reagents [1]–[3]. The development of enzymes for research or industrial purposes has depended heavily on the use of microbial sources because microbes can be produced economically in short fermentations and inexpensive media [4]. Among microbes, extremophiles were recognized as a source for novel enzymes potentially associated with enhanced properties [5]. Traditionally, discovery of novel enzymes from microbes comprised screening for the microbe, enzyme isolation and characterization, followed by cloning of selected enzymes to produce overexpression systems [6]. The bottleneck of the traditional microbial screening for novel enzymes is the fact that less than 1% of environmental bacteria can be cultivated through standard laboratory techniques [7]. Metagenomics has appeared as an alternative approach to conventional screening. By directly cloning environmental DNA (or metagenome) in a proper host, the metagenome can be screened even if the source organisms cannot be cultured [8], [9]. This approach involves using conventional basic local alignment search tool (BLAST) searches [10] against protein databases such as the non-redundant NCBI database or UniProt [11]. Enzymes can then be identified from the resulting hits.

Phenylalanine ammonia-lyase (PAL; EC 4.3.1.24 and EC 4.3.1.25) catalyzes the non-oxidative deamination of l-phenylalanine into (*E*)-cinnamic acid. PALs are essential in plants at the starting point of the phenylpropanoid pathway, catalyzing the first step in the biosynthesis of multiple phenylpropanoids, such as lignins, flavonoids and coumarins. PAL enzymes are encoded by a family of genes and the presence of PAL isoforms is common in higher plants [12]. It has been suggested that the phenylpropanoid metabolism is modulated and PAL is probably the rate-limiting enzyme in this pathway [13]. Because of its central role in plant metabolism, PAL is one of the most thoroughly studied plant enzymes and is a potential target for herbicides [14]. Feedback inhibition of PAL activity by its own product, (*E*)-cinnamic acid, was demonstrated *in vitro* [15] and it was also proposed that (*E*)-cinnamic acid regulates transcription of PAL genes *in vivo* [16]. In addition to plants, presence of PAL was also reported in fungi and in some bacteria [17].

**Figure 1:**
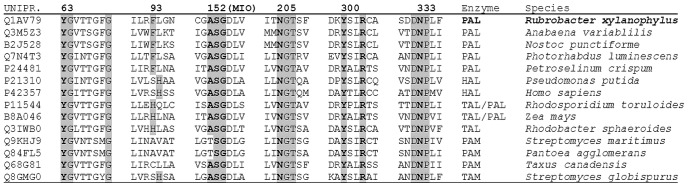
Characteristic amino acid sequence motifs of *Rx*PAL compared to sequence motifs of other aromatic amino acid ammonia-lyases and aminomutases. Numbering above motif columns refer to the sequence of *Rx*PAL. The characteristic tripeptide ASG involved in formation of the MIO moiety is indicated by (MIO) at the top of the alignment. The alignment was constructed using sequences of ammonia-lyases (PAL, HAL, TAL) as well as aminomutases (PAM, TAM) from different eukaryotic and prokaryotic sources.

PAL belongs to the 3,5-dihydro-5-methylidene-4*H*-imidazol-4-one (MIO)-containing ammonia-lyase family, together with histidine ammonia-lyase (HAL, EC 4.3.1.3) and tyrosine ammonia-lyase (TAL, EC 4.3.1.23). These latter enzymes catalyze the deamination of the corresponding l-amino acids (HAL: L-His; TAL: L-Tyr) [18], [19]. The family of ammonia-lyases show a strong similarity to the family of aminomutase enzymes (l-phenylalanine and l-tyrosine 2,3-aminomutases, PAM [20]–[23] and TAM [24], [25], respectively) as indicated by presence of the unusual catalytic MIO moiety in both ammonia-lyases and these aminomutases and also by the fact that both PAM [20] and TAM [26] have ammonia-lyase activity. The amino acid residues involved in formation of the MIO moiety constitute a strictly conserved tripeptide of alanine, serine and glycine (ASG, see Figure 1). Mutation of serine 143 in HAL from *Pseudomonas putida* [27] and serine 203 in PAL from parsley (*Petroselinum crispum*) [28] to alanine decreased the activity more than a thousand fold [28]. Mutation of other conserved serines had little or no effect.

There are several potential applications of PALs (Table 1) [17], [18]. In addition to the known therapeutic enzymes [29], a sufficiently stable PAL protein may be used as a potential therapeutic enzyme in cancer treatment as shown by *in vitro* and *in vivo* (in mice) experiments [30]–[34]. The potential of chemically modified PALs was also considered in enzyme replacement therapy of phenylketonuria (PKU) [35], [36]. In 2011, BioMarin Pharmaceutical has announced that a PEG-PAL product (PEGylated recombinant PAL) for the treatment of PKU is currently in Phase II clinical trials.

**Table 1:** Application of phenylalanine ammonia-lyases.

Area of application	Reference
Target enzyme for herbicides	[14]
Therapeutic enzyme in cancer treatment	[30]–[34]
Enzyme replacement therapy of phenylketonuria	[35,36]
Biocatalyst for preparation of various l- and d-α-amino acids	[18], [19], [39]–[45]

In addition to medical applications, PAL has synthetic potentials as a biocatalyst. Due to increasing consumption of the artificial sweetener aspartame (an aspartic acid-phenylalanine dipeptide), a large scale production scheme for producing l-phenylalanine was developed that relies on addition of ammonia to (*E*)-cinnamic acid and uses PAL enzyme as the biocatalyst [37], [38]. PALs of plant and yeast origin were also useful as biocatalysts in the preparation of various unnatural l- and d-α-amino acids [18], [19], [39]–[45]. For example, DSM Pharma Chemicals developed a large scale enantioselective synthesis of (*S*)-2-indolinecarboxylic acid using PAL biocatalysis in the key step [46].

Despite their potential significance, only a few bacterial PAL enzymes has been isolated and characterized to date (from the following organisms: *Streptomyces verticillatus* [47], *Streptomyces maritimus* [48], *Photorhabdus luminescens* [49], and two cyanobacteria: *Anabaena variabilis* [35], [50], *Nostoc punctiforme* [50]). The rarity of PAL in bacteria may be explained by the fact that phenylpropanoids rarely occur in these organisms. However, bacterial PALs seem to be involved in biosynthesis of special bacterial products such as the enterocin antibiotics by *S. maritimus* [48] and 3,5-dihydroxy-4-isopropylstilbene by *P. luminescens* [49] that use (*E*)-cinnamic acid, the product of the PAL-catalyzed reaction, as precursor.

Structural studies on PALs showed that they exist as homotetramers possessing a conserved polypeptide chain fold. A characteristic difference between prokaryotic [35], [50] and eukaryotic PAL [12], [51], [52] enzymes is the presence of an approximately 120-residue long *C*-terminal multi-helix domain that is found only in eukaryotic PAL proteins. This domain forms an arch over the active site and it was proposed to function as a shielding domain restricting substrate entry and product exit [12]. Alternatively, it was hypothesized that a role of this C-terminal extension is to decrease the lifetime of eukaryotic PALs by destabilizing the conformation of a conserved Tyr110 (*Petroselinum crispum* PAL, *Pc*PAL) lid loop [53]. On that basis it might be assumed that prokaryotic PALs [35], [50], [54] are more thermostable than their eukaryotic analogues [12], [51], [52].

A comprehensive study comprising more than twenty enzymes including PAL from *Rhodotorula glutinis* (*Rg*PAL) indicated that their thermal stability was correlated (albeit weakly) with the growth temperature of the source organism [55]. The tentative higher thermostability of bacterial PALs was also supported by investigations of the enzyme coded by EncP gene of the thermotolerant marine bacterium *Streptomyces maritimus.* This protein was shown to function as a PAL at 30°C [48], [54] and its PAL activity increased exponentially from 30 to 64°C, reaching a maximum activity at 74°C [54]. The enzyme encoded by the gene AdmH of the mesophilic bacterium *Pantoea agglomerans* is a phenylalanine 2,3-aminomutase (*Pa*PAM) which provides (*S*)-β-phenylalanine required for the biosynthesis of the antibiotic andrimid [56], [57]. Unexpectedly, at elevated temperature enzyme AdmH exhibited thermophilic PAL activity similar to EncP [54].

Our goal in this study was to identify novel PALs in thermophilic bacteria among the hits of BLAST searches against the non-redundant NCBI and UniProt databases. Accordingly, we cloned the synthetic gene from thermotolerant bacterium *Rubrobacter xylanophilus* (*Rx*PAL) and characterized the encoded *Rx*PAL enzyme. The enzymatic properties of *Rx*PAL were determined at different pH values and a point mutation was also constructed within the characteristic ASG tripeptide involved in formation of the MIO moiety. The mutant protein was investigated by differential UV spectroscopy and data indicated loss of the MIO moiety as a result of the mutation. Construction of a structural model allowed insights into the structural basis of increased alkaline tolerance of *Rx*PAL.

## Materials and Methods

### Identification of the gene encoding a phenylalanine ammonia-lyase in the thermophilic bacterium *Rubrobacter xylanophilus*

BLASTp search against the non-redundant NCBI protein database using the sequence of PAL from *Photorhabdus luminescens* (*Pl*PAL) [49], [58] (UniProt code: Q7N4T3) resulted in a potential hit (Acc. code: YP_644511.1, encoding 540 AA) denoted as putative phenylalanine/histidine ammonia-lyase of the thermophilic bacterium *Rubrobacter xylanophilus* DSM 9941. BLASTp search against the Bacteria subsection of UniProt database using the *Pl*PAL sequence resulted in a potential hit (UniProt code: Q1AV79, encoding 540 AA) referring to the same gene (Acc. code: YP_644511.1, cf. Fig. 1).

### Cloning, expression and purification of *Rx*PAL

The gene of the *Rubrobacter xylanophilus* PAL (NCBI acc. code: YP_644511.1, UniProt code: Q1AV79, encoding 540 AA) was optimized to the codon usage of *E. coli*. The 1632 bps long synthetic gene insert was excised from the carrier pMK plasmid via *Eco*RI and *Xho*I restriction digests. The gene fragment was separated from the vector DNA using agarose gel electrophoresis. The purified insert was then directionally ligated into the pBAD-HisB expression vector. Results of cloning were confirmed by sequencing using the following forward and reverse primers: 5′–CCTGACGCTTTTTATCGCAACTC–3′ and 5′–GAGGCATCCTGGTACCCCAG–3′, respectively.

A *rec*A, *end*A, *ara*BADC(−) and *ara*EFGH(+) TOP10 *E. coli* strain, which was able to transport l-arabinose without metabolizing it, was used for expression of the *Rx*PAL protein. The usual CaCl_2_/MgCl_2_ transformation protocol [59], [60] was used for transforming *E. coli* TOP 10 strain with the plasmid pBAD-HisB-*Rx*PAL. For stages 1 and 2 of the transformation protocol, buffers TFB I and II were used, respectively [TFB I: pH 5.8 (pH adjusted with 10% acetic acid), 100 mM RbCl, 50 mM MnCl_2_, 30 mm potassium acetate, 10 mM CaCl_2_, 15% glycerol. Store at 4°C; TFB II: pH 6.8 (pH adjusted with 1 M KOH), 10 mM MOPS, 10 mM RbCl, 75 mM CaCl_2_, 15% glycerol.]

Sterile LB medium (50 ml) containing ampicillin (100 µg ml^−1^) was inoculated with the transformed *E. coli* TOP 10 cells. The culture was shaken at 220 rpm at 37°C until the OD_600_ rose to 1–2 (ca. 12 h). Subsequently, a 0.5 ml inoculum from the transformation culture was transferred into sterile LB medium (500 ml) containing ampicillin (100 µg ml^−1^). The culture was shaken at 220 rpm at 37°C until the OD_600_ rose to ∼0.4–0.6. Then the temperature was decreased to 25°C and the cells were induced by addition of 0.02% l-arabinose. The culture was shaken at 220 rpm at 25°C for further 16 h. The cells were harvested by centrifugation of the cell-suspension at 3000×g. All of the subsequent procedures were carried out on ice-bath.

The pellets were resuspended in 5 ml lysis buffer (150 mM NaCl, 50 mM TRIS pH 8.0, 10 mM BME, protease inhibitor cocktail: 2 mM PMSF and 5 mM BA) and the cell suspension was sonicated (3×45 sec) at amplitude 40% and pulsation 60% using a Bandelin Sonopuls HD 2070 instrument. Sonication was performed until the viscosity of the suspension significantly decreased. The extract was centrifuged at 5000×g for 30 min and the supernatant was used for further purification.

**Figure 2:**
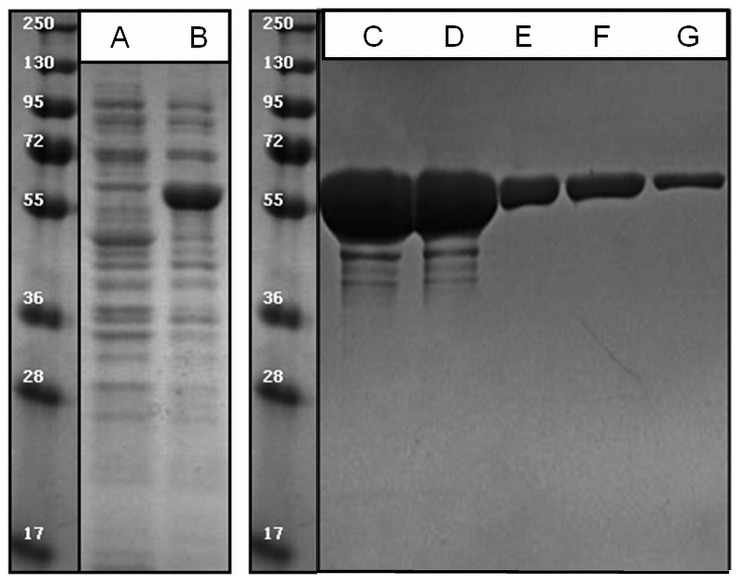
Expression and purification of *Rx*PAL. Bacterial cells transformed with the pBAD-HisB plasmid containing the gene for *Rx*PAL were extracted and the cell extract was run on 12% SDS-PAGE gels. Left panel shows expression pattern before induction (Lane A) and 10 hours after induction (Lane B). Right panel shows purified *Rx*PAL protein (Lane C: 300 µg, Lane D: 200 µg, Lane E: 80 µg, Lane F: 40 µg, Lane G: 20 µg).

The recombinant *Rx*PAL carrying *N*-terminal His_6_-affinity tag was purified on a Ni–NTA affinity chromatography column (Qiagen, Germany) according to the manufacturer's protocol using 45–60 mg soluble cell protein per ml Ni-NTA agarose and elution with 500 mM imidazole buffer (500 mM imidazole in Low Salt Buffer, pH 7.5). The resulting eluate was dialyzed against 1000 ml of 50 mM PBS (I = 300 mM, adjusted with KCl, 5 mM BME) per 5 ml eluate. SDS-PAGE investigation of the product indicated that the purified PAL had a high degree of purity (cf. Fig. 2, purity >95%; note that even in overloaded gel lanes contamination with other proteins was minor).

### Creation of the S153A point mutant of *Rx*PAL

The S153A mutant of *Rx*PAL was constructed by following the instruction manual of QuickChange site-directed mutagenesis kit [61]. For mutagenesis reactions S153A-forward 5′–GCGTGGAAGTTGTGGTGCTGCTGGTGATCTGGTTCCTCTGTC–3′ and S153A-reverse 5′–GACAGAGGAACCAGATCACCAGCAGCACCACAACTTCCACGC–3′ oligonucleotides were used as primers. Mutation was confirmed by standard sequencing using pBAD-forward 5′–ATGCCATAGCATTTTTATCC–3′ and pBAD-reverse 5′–GATTTAATCTGTATCAGG–3′ primers. The mutant protein was expressed and purified as the wild type.

### Phenylalanine ammonia-lyase activity assay for *Rx*PAL

In the assay to determine enzymatic activity of *Rx*PAL under different circumstances, we used the previously published method that relies on the spectral differences between the substrate l-phenylalanine and the product (*E*)-cinnamic acid [62]. The absorption of (*E*)-cinnamic acid at 290 nm is characteristically higher than that of l-phenylalanine, hence we followed the enzymatic reaction in a spectrophotometer at 290 nm. Progress of ammonia elimination from l-phenylalanine was monitored by detection of (*E*)-cinnamic acid production at 290 nm (ε_290_ = 10^4^ M^−1^ cm^−1^ at 25°C) in thermostatted, standard UV cuvettes of 1 cm optical path length in a Specord 200 spectrophotometer. Purified *Rx*PAL (2 µM: 50 µl of a 2.2 mg ml^−1^ solution) was added to the buffer (500 µl; pH changed in 0.5 steps between 4.0–12 by using 0.1 M buffers with ion strength kept at 250 mM) containing 20 mM l-phenylalanine [pH 4.0–6.0: NaOAc buffer, pH 6.0–7.0: Tris-Bis/Tris-HCl buffer, pH 9.0–10.0: Sodium phosphate buffer, pH 10.0–11.5: CAPS buffer, pH 12.0: piperidine] and the absorption increase at 290 nm was recorded for 10 min.

### Circular dichroism (CD) spectra of *Rx*PAL in the pH 6.5–12 range

Aliquots of purified *Rx*PAL (concentrations 0.5 mg ml^−1^ in the far UV, and 2 mg ml^−1^ respectively for CD measurements in the near UV) were thermostatted at 20°C in different buffers between pH 6.5–12.0 (0.1 M buffers with constant ion strength kept at 250 mM; pH 6.5–7.0: Tris-Bis/Tris-HCl buffer, pH 9.0–10.0: Sodium phosphate buffer, pH 10.0–11.5: CAPS buffer, pH 12.0: piperidine) for 15 min and then the CD spectra were recorded on a JASCO J-720 spectropolarimeter [63]–[65]. The path length of the cuvettes used for the far UV and near UV measurements was 0.1 and 1 cm, respectively.

### Identification of the MIO group in UV difference spectra of *Rx*PAL and its S153A mutant

UV difference spectra of *Rx*PAL and the mutant variant S153A (expected to lack the MIO moiety) were recorded at various *Rx*PAL concentrations (0.1–2.2 mg mL^−1^) at 20°C in different buffers between pH 6.5–12.0 (1 ml; 0.1 M buffers with constant ion strength kept at 250 mM; pH 6.5–7.0: Tris-Bis/Tris-HCl buffer, pH 9.0–10.0: Sodium phosphate buffer, pH 10.0–11.5: CAPS buffer, pH 12.0: piperidine) from 240 to 360 nm using 1 cm quartz cuvettes in a dual-beam Specord 200 spectrophotometer. The blank experiment contained the MIO-less S153A mutant *Rx*PAL protein lacking the essential 4-methylidene-imidazolon-5-one prosthetic group at the same concentrations.

### Homology model of *Rx*PAL

To get insight into the structure of PAL from *Rubrobacter xylanophilus*, a homology model was generated with MODELLER [66]–[69] using PAL from *Anabaena variabilis* (UniProt code Q3M5Z3; PDB code 3CZO [35]; 35% identity with BLOSUM62 matrix) as the template. The raw model was refined with the MacroModel [70] module of Schrödinger Suite 2012 (implicit water solvent model, OPLS2005 force field, threshold 0.1 kcal mol^−1^). Poisson-Boltzmann electrostatic potential surfaces were created with Maestro [71] with default settings. Salt bridges were evaluated with VMD [72] by assuming ion-pairs between residues with oxygen-nitrogen distance within 3.2 Å. Residues participating in multiple salt bridges were counted only once when proportion of amino acids involved in salt bridge formation was determined.

## Results and Discussion

### Identification and expression of *Rx*PAL

Bioinformatics approach based on BLAST searches [10] for sequences similar to the sequence of PAL from *Photorhabdus luminescens* [49], [58] against non-redundant protein databases such as the UniProt [11] resulted in several sequence hits. The potential PAL candidates were distinguished from the numerous histidine ammonia-lyases (HALs) by Clustal W multiple sequence alignments [73] implemented in UniProt. The presence of Phe and Leu in the positions analogous to Ser83-His84 in the sequence of the known histidine ammonia-lyase from *Pseudomonas putida* (*Pp*HAL; UniProt code: P21310) [74], [75] was characteristic to *Pl*PAL (UniProt code: Q7N4T3, encoding 540 AA) and to the putative phenylalanine ammonia-lyase of the thermophilic bacterium *Rubrobacter xylanophilus* DSM 9941 (UniProt code: Q1AV79) as well (Fig. 1). Moreover, this important ^93^FL sequence characterizing the aromatic binding region part of genuine PALs in the putative *Rx*PAL sequence was dissimilar to that of the SH motif of HALs and HL or HQ motif of TALs at similar positions (Fig. 1).

The first strain of the genus *Rubrobacter* was isolated from gamma-irradiated hot spring water [76]. This species, *R. radiotolerans* was slightly thermophilic with an optimum growth temperature of about 45°C. Later, a true thermophilic strain with an optimum growth temperature of about 60°C was isolated from a hot runoff of a carpet factory and was identified and named as a new species *R. xylanophilus* [77]. In the present study, we cloned, expressed and characterized a PAL from this Gram-positive, thermophilic and radiotolerant bacterium strain after identifying putative PAL-encoding gene by screening the genomes of bacteria for members of the aromatic amino acid ammonia-lyase-family online with the programs BLAST and Clustal W using all parameters set to their default values. To our knowledge, no PAL enzyme has been characterized from this thermophilic bacterium. Having identified the putative PAL-coding gene in *R. xylanophilus*, this gene was synthesized with an optimized codon usage for *E. coli* host strains. Expression and purification of *Rx*PAL from *E. coli* host was successful and the resulting preparation showed high degree of electrophoretic purity (Fig. 2).

### *Rx*PAL has an extremely alkaline pH optimum for catalysis

**Figure 3:**
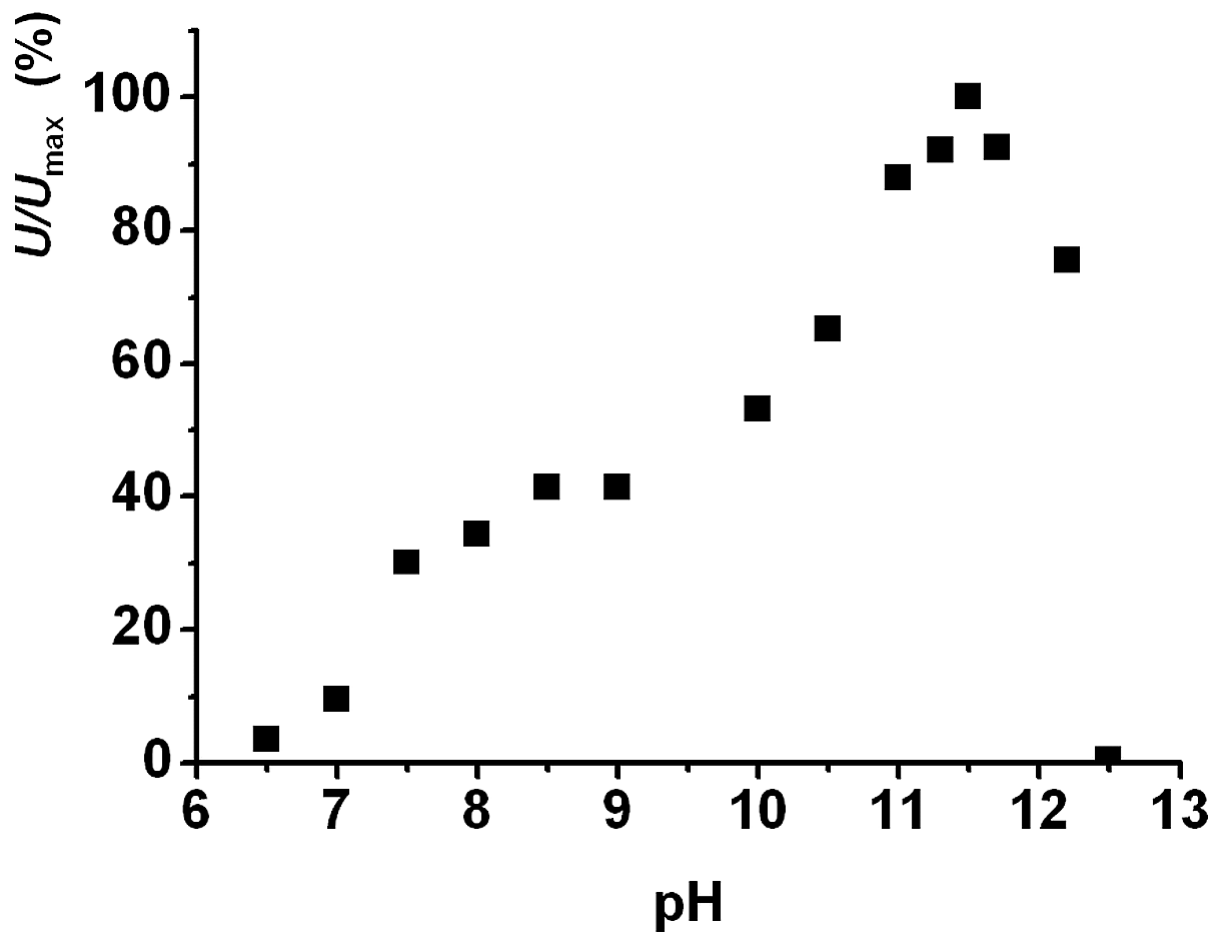
Catalytic optimum for *Rx*PAL is in the high alkaline range. PAL activity was measured in triplicates at the different pH values, data represent average (standard deviation was <15%). Measurements were performed by following production of (E)-cinnamic acid at 290 nm in a spectrophotometer (cf. Materials and methods for more details). Data are presented as relative to the maximal activity observed at pH 11.5. Data indicate a local pH optimum around pH of 8.5, and a global optimum at pH 11.5.

The enzymatic activity of PAL was monitored between pH 6.5–12.0 (Fig. 3). At each pH values, measurements were carried out in independent triplicates, and the data showed less than 15% standard deviation. pH values lower than 6.5 were not tested because of the precipitation of the protein. The rate of the PAL catalyzed reaction slowly increased up to pH 8.5. At higher pH values, enzyme activity was increasing further and reaching a maximum at pH 11.5. Up to pH 11.8, enzymatic activity was still retained but at higher pH values it abruptly dropped, probably due to protein denaturation. PAL activity was fully stable at all pH≤11.5 for 1 h at room temperature. The pH range of the phenylalanine ammonia-lyases characterized so far is clearly on the alkaline range, with a pH optimum of 8.5–9.5. In contrast to other PALs, however, activity and stability of the *Rx*PAL was shown to be significantly higher at a strongly alkaline pH (around 11), rendering the new enzyme attractive as a biocatalyst under these conditions (Fig. 3). The observed high activity at elevated pH values is especially useful regarding the reverse reaction, wherein ammonia addition to achiral arylacrylates resulting enantiomerically pure l-configured unnatural α-amino acids in the presence of 5–6 M NH_3_/NH_4_^+^ in the pH 10–11 range [18], [19], [40], [41].

### Circular dichroism spectra argue for high amount of α-helical secondary structural elements and support alkaline resistance

**Figure 4:**
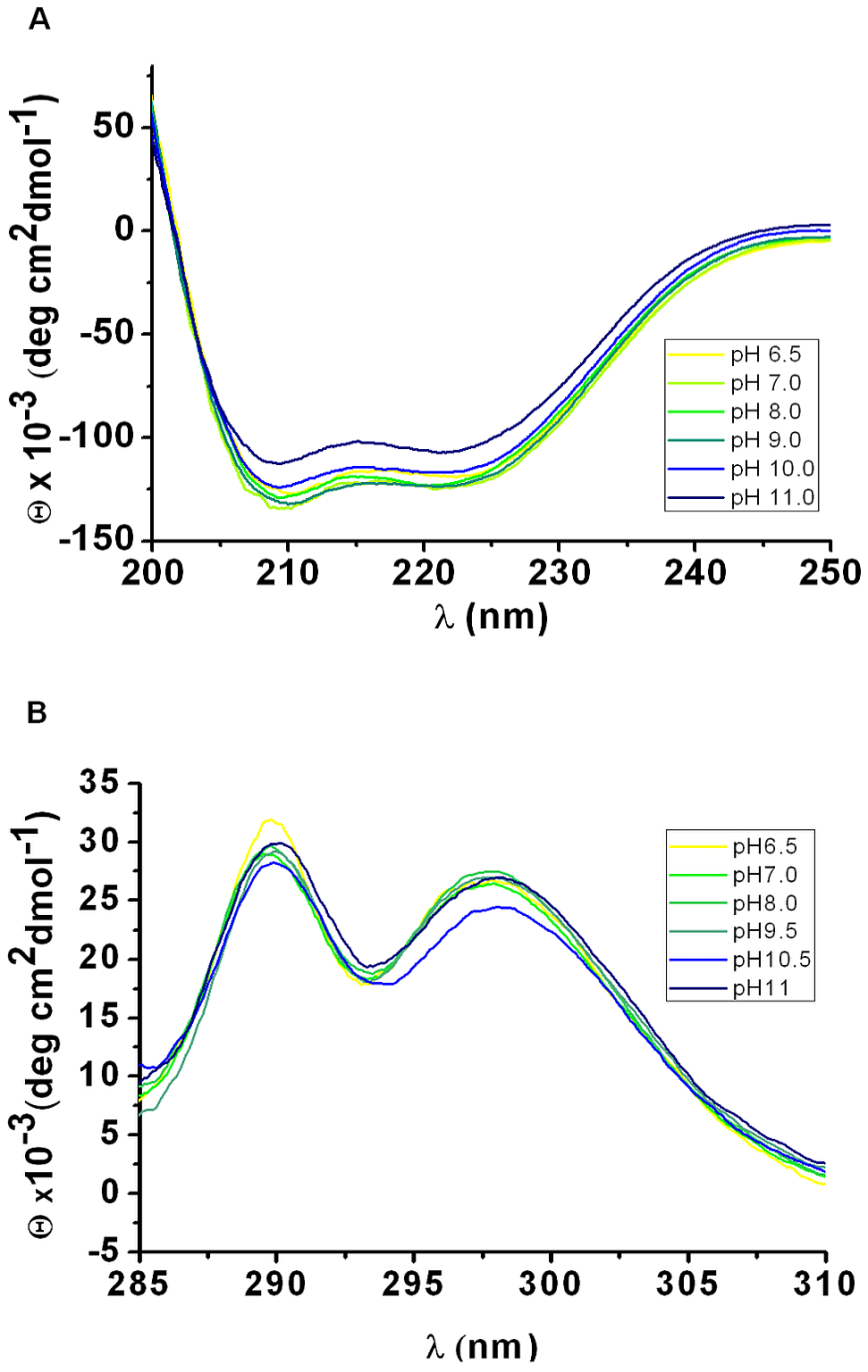
Structural integrity of *Rx*PAL is well preserved at high pH values. Circular dichroism spectra of *Rx*PAL were recorded in the far UV (195–250 nm) (Panel A) and near UV (285–310 nm) (Panel B) wavelength range, at different pH values.

To investigate the proportions of different secondary structural elements in *Rx*PAL, far UV CD spectroscopy was applied (Fig. 4A). The far UV CD spectra clearly indicated that *Rx*PAL is associated with high content of α-helical secondary structures, as the spectra nicely show the corresponding characteristic double maxima at 208 and 222 nm. Using the K2d software [78], [79], we could estimate that *Rx*PAL protein possesses 88% α-helices, 7% β-sheets, and 5% random coils. These values are only indicative but it is important to note that they are in good agreement with secondary structural content of other PAL enzymes, for which a three-dimensional structure was already determined by X-ray crystallography. The far UV CD spectra measured at the different pH values retained the characteristics of the double maxima at 208 and 222 nm wavelength values. Hence, based on the far UV CD spectra (cf. Fig. 4A), we conclude that the overall secondary structure of *Rx*PAL is well preserved up to pH 11.0.

Near UV CD measurements were also carried out as the fine spectral details in this wavelength range are diagnostic for conformational changes. Fig. 4B show that there are two major peaks in the near UV CD spectrum of *Rx*PAL, at 289 and 298 nm, characteristic for tryptophan or tyrosine residues, respectively. Tryptophan residues are usually associated with peaks around 290 nm with a fine structure between 290 and 305 nm, whereas tyrosine residues are usually characterized with peaks between 275 and 282 nm, but the fine structure at longer wavelengths may be obscured by those from tryptophan. Both of these major peaks are well observable in the protein spectrum up to pH 11.0, although at this pH, the relative height of the two peaks are somewhat altered: the peak at the higher wavelength, putatively associated with tryptophan residues is smaller and also shows a slight red-shift. The spectral characteristic of tryptophan residues are especially sensitive to changes in the protein microenvironment. These spectra provided additional convincing information on the integrity of the tertiary structure between pH 6.5–11.0, confirming the stability of the enzyme at highly alkaline pH (Fig. 4B), and also showed that at pH 11.0 some slight conformational changes may already be initiated. At pH 12.5, CD spectra (data not shown) indicated the partial denaturation of the protein in agreement with the results of activity measurements.

### Monitoring the presence of the catalytically essential MIO group in the structure of *Rx*PAL

**Figure 5:**
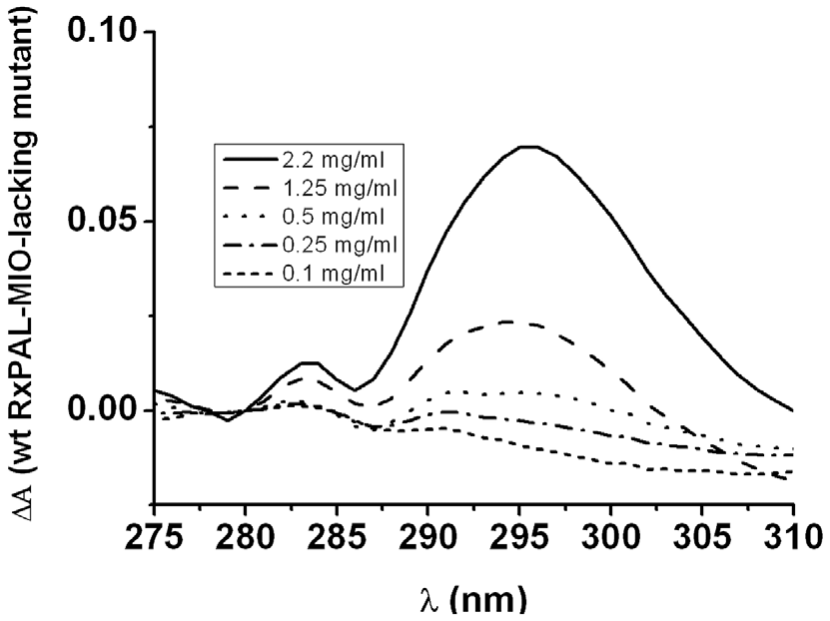
Mutation of wt *Rx*PAL at the putative MIO site erases the MIO-specific spectroscopic signal. Difference spectra for wild type and S153A mutant *Rx*PALs were recorded at the indicated protein concentrations. The difference spectra were recorded by placing the wild type protein in the control cuvette and the mutant protein in the sample measuring cuvette at the respective concentrations in the dual-beam spectrophotometer. Difference spectra show the presence of the MIO-characteristic spectroscopic signal only in the wild type enzyme, but not in the mutant.

The catalytically essential MIO moiety in PAL enzymes was shown to generate a characteristic peak in the near-UV domain [80]. The amino acid sequence of *Rx*PAL contains the ^152^ASG^154^ sequence motif that was shown to be involved in the formation of the MIO group in other PALs. To investigate the presence and function of this motif in *Rx*PAL, we produced the S153A mutant, in which the serine residue important for MIO formation was replaced by alanine, disrupting thereby MIO formation. The S153A *Rx*PAL mutant was assayed and showed practically total loss of enzymatic activity in eliminative deamination of l-Phe. UV spectra of the wild-type *Rx*PAL and of its S153A mutant lacking the MIO group were investigated to prove the presence and to estimate the amount of this group in this highly alkalophilic enzyme (Fig. 5). Results clearly showed that the wild type *Rx*PAL possessed the MIO-characteristic UV absorbance peak being absent in the spectrum of the S153A mutant. The presence of the catalytically essential MIO in *Rx*PAL was determined by UV-difference spectra of the active enzyme and the S153A mutant lacking MIO at several pH values between 6.5–11.0 (data not shown). This fact provided a direct spectroscopic evidence for the formation of the MIO group with involvement of the ^152^ASG^154^ sequence segment in *Rx*PAL.

### Homology modeling rationalization of the thermotolerance and alkalophilicity of *Rx*PAL

**Figure 6:**
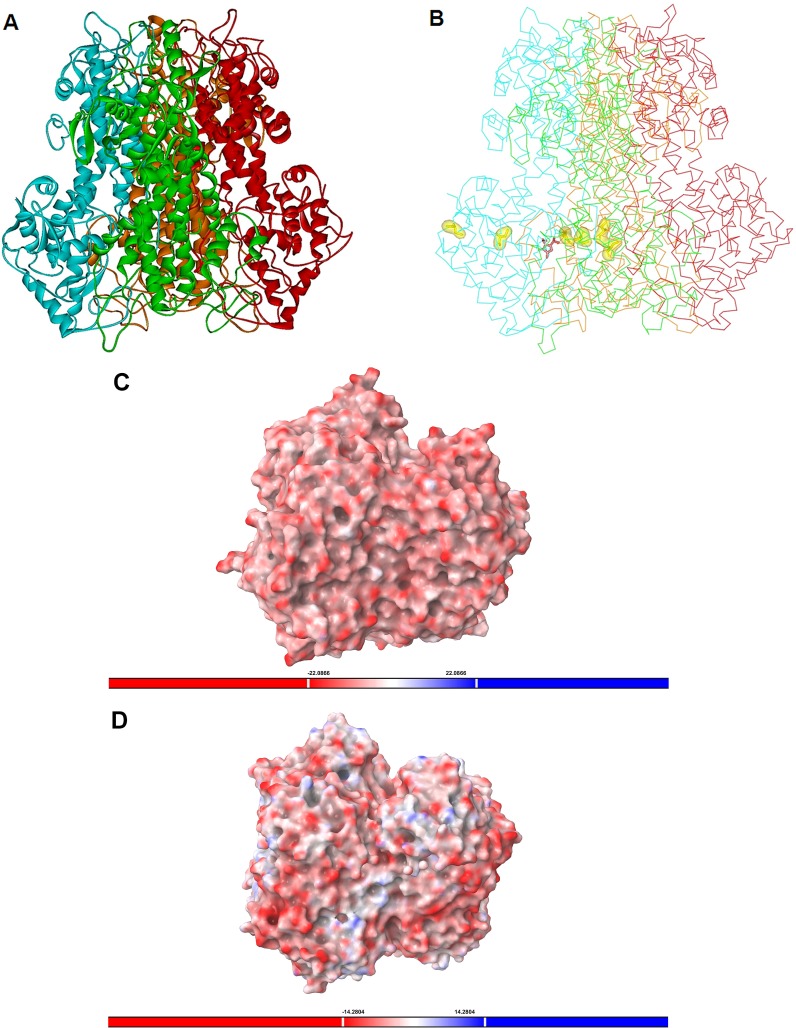
Structural properties of *Rx*PAL by homology modeling. Ribbon representation of the homotetrameric model of *Rx*PAL (Panel A) and backbone line representation model with the MIO (in pink) surrounded by three pairs of cysteines (in yellow) which may form two intra-subunit and one inter-subunit disulfide bridges (Panel B). The electrostatic surface potential representation of *Rx*PAL (Panel C) compared to that of less alkalophilic *Av*PAL (Panel D, PDB code 3CZO [35]) indicated highly negatively charged *Rx*PAL surface.

For explanation at the molecular level of the high alkalophilicity of the PAL from *Rubrobacter xylanophilus* and its thermotolerance (e.g. the *k*_cat_ = 0.1 s^−1^ catalytic rate constant of the l-Phe conversion at 20°C and pH 8.8 increased ten times to *k*_cat_ = 1.0 s^−1^ at 45°C), a homology model was generated for *Rx*PAL using the experimental structure of PAL from *Anabaena variabilis* (PDB code 3CZO [35]; 35% identity) as the template. The model of *Rx*PAL (Fig. 6A) showed several putative disulfide bonds surrounding the active site (Fig. 6B) and unusually high number of salt bridges (Table 2) that may explain the thermotolerance of this enzyme.

**Table 2:** Ionizable amino acid composition, negative charge excess and salt bridge content of *Rx*PAL compared to other aromatic amino acid ammonia-lyases.

Taxonomy	Enzyme^a^	Residue/chain	CYS/chain	ASP (%)	GLU (%)	LYS (%)	ARG (%)	Acid excess	Salt bridges^a^	AA in SB (%)
*Rubrobacter xylanophilus*	PAL	540	9	4.8	8.5	2	8.3	16	285	23.1
*Photorhabdus luminescens*	PAL	532	7	4.5	6.2	5.1	3.9	9	220	16.0
*Pseudomonas putida*	HAL	509	7	5.1	5.7	3.5	5.3	10	128	11.4
*Petroselinum crispum*	PAL	716	9	3.8	7.7	5.6	5.6	12	148	9.1
*Rhodosporidium toruloides*	PAL	716	4	4.2	5.7	4	4.9	7	137	8.0
*Rhodobacter sphaeroides*	TAL	523	8	5.5	4.2	1	8.8	0	98	8.2
*Anabaena variabilis*	PAL	567	6	5.5	3.4	3.2	4.4	7	74	5.9
*Nostoc punctiforme*	PAL	569	7	5.6	3.9	3.1	4.4	11	68	5.3

a 3D structures used for salt bridge-content evaluation: *Rx*PAL (homology model from this work), *Pl*PAL (homology model from ref. [55]), *Rt*PAL (PDB code: 1Y2M), *Pp*HAL (PDB code: 1GKM), *Pc*PAL (PDB code: 1W27), *Rs*TAL (PDB code: 2O7E), *Av*PAL (PDB code: 3CZO), *Np*PAL (PDB code: 2NYF).

A comparison of structural basis for thermal stability in archaeal and bacterial proteins revealed that increased salt bridge and Glu content are among the important stabilizing factors of heat-resistant bacterial proteins [81]. Most thermophilic proteins tend to have more salt bridges, and achieve higher thermostability by up-shifting and broadening their protein stability curves. The enhanced salt bridge content reduces ΔC_p_ (i.e. the change in heat capacity upon unfolding) and increases ΔG_unfold_ (the change in the Gibbs free energy upon unfolding) and thereby stabilizes the protein at high temperatures [82], [83]. Therefore, a comprehensive comparison of several aromatic amino acid ammonia-lyases including *Rx*PAL was carried out based on their charged amino acid, salt bridge and cysteine content. The salt bridge-content of homotetrameric aromatic amino acid ammonia-lyase structures was evaluated by VMD [72] (Table 2).

Data in Table 2 indicate that *Rx*PAL has the highest number of salt bridges (285) and highest proportion of amino acids involved in salt bridge formation (23.1%) among all listed ammonia-lyases including HAL of *Pseudomonas putida* (*Pp*HAL) which tolerates a heat shock around 70°C for a few minutes during its purification [27] but contains less salt bridges (128) and lower proportion of amino acids involved in salt bridge formation (11.4%). Note, that no disulfide bond was identified in crystal structure of *Pp*HAL [75]. Among the PALs characterized so far *Pc*PAL and *Rt*PAL have relatively high thermostability (temperature optima: 58°C for *Pc*PAL at pH 8.5 [15], 50°C for *Rt*PAL at pH 8.5 [34]) which correlates also with the relatively high number of salt bridges (*Pc*PAL: 148; *Rt*PAL: 137) and high proportion of amino acids involved in salt bridge formation (*Pc*PAL: 9.1%; *Rt*PAL: 8.0%). The high number of salt bridges (220) in the 3D model of *Pl*PAL and the high proportion of amino acids involved in salt bridge formation (16.0%) predicts *Pl*PAL also as thermostable. All the other mesophilic PALs and TAL (*Av*PAL, *Np*PAL and *Rs*TAL) contain significantly fewer salt bridges.

Disulfide bonds can also affect the thermostability of proteins. A study of the thermophilic *Thermomyces lanuginosus* xylanase indicated that a disulfide bridge introduced into the *N*-terminal region of the enzyme shifted the apparent temperature optimum at pH 6.5 upwards by about 10°C to 75°C [84]. Structural investigation of a short-chain alcohol dehydrogenase from the hyperthermophilic archaeon *Thermococcus sibiricus* indicated that in case of a tetrameric structure it is intersubunit disulfide bond as well as a large number of surface ion pairs which may contribute to its thermotolerance [85]. Studies with *Photinus pyralis* firefly luciferase showed that disulfide bridges either near or remote to the active site contributed to the thermostability but the one near to the active site region had more impact on kinetic characteristics of the enzyme [86]. Moreover, disulfide bridges contributed to the enhanced pH stability of the protein at the alkaline region as well.

Because disulfide bonds may also affect the thermostability of proteins, putative disulfide bridges within *Rx*PAL were evaluated by inspection of the critical distances (<6 Å) between cysteines within the homology model (Fig. 6B). In the vicinity of the MIO-prosthetic group 35C(A)-116C(A), 150C(A)-420C(C) and 231C(B)-478C(B) were identified as possible disulfide bonds. Note, that 35C(A)-116C(A) SS bond could fix the *N*-terminal region to the main structure and 150C(A)-420C(C) would be an intersubunit disulfide bond between two monomers of the tetrameric structure.

A crystal structure study of an endoxylanase from an alkalophilic *Bacillus* sp. NG-27 (optimally active and stable at 70°C and at a pH of 8.4) when compared with other alkalophilic xylanases suggested that a protein surface rich in acidic residues may be an important common feature of these alkalophilic thermostable enzymes [87]. Another study on the β-mannanase from the alkalophilic *Bacillus* sp. N16-5 with a pH optimum of enzymatic activity at pH 9.5 explained the alkalophilicity of this enzyme by the high number of negatively charged residues and fewer polar residues exposed to the solvent on the enzyme surface [88].

Data in Table 2 showed that among the ammonia lyases listed, *Rx*PAL had the highest proportion of amino acids involved in salt bridge formation (23.1%) and the largest excess of acidic residues (16). Moreover, the comparison of electrostatic surface potential of the alkalophilic *Rx*PAL structure (Fig. 6C, pH optimum 11.5) to that of the more neutral *Av*PAL (Fig. 6D, pH optimum 7.5 [35]) indicated an almost uniform negative charge coverage of the alkalophilic *Rx*PAL (Fig. 6C) whereas both negatively and positively charged surfaces were visible for the near neutral *Av*PAL (Fig. 6D).

Note, that high enzymatic activity of PALs at elevated pH (pH 10–11 in 5–6 M NH_3_/NH_4_^+^) was required in applications of PALs as biocatalyst to prepare enantiomerically pure l-configured α-amino acids by stereoselective addition of ammonia to achiral arylacrylates [18], [19], [40], [41].

## Conclusion

Bioinformatics' tools proved to be useful for the identification of novel PALs from thermotolerant bacteria, as we demonstrated by the recognition, expression and characterization of a novel PAL of the true thermophile *Rubrobacter xylanophilus* (*Rx*PAL). Homology modeling and bioinformatics based analyses were also used to explain the thermotolerance and high alkalophilicity of the novel *Rx*PAL. Based on its thermophilic and highly alkalophilic nature *Rx*PAL has a potential to be exploited as biocatalyst in stereoselective synthetic biotransformations under extreme conditions.
